# Discrimination of Dengue Diseases in Children Using Surface-Enhanced Raman Spectroscopy Coupled with Machine Learning Approaches

**DOI:** 10.1021/acs.analchem.5c01182

**Published:** 2025-07-07

**Authors:** Uraiwan Waiwijit, Pitak Eiamchai, Saksorn Limwichean, Mati Horprathum, Tanapan Prommool, Chunya Puttikhunt, Adisak Songjaeng, Nuttapong Kaewjiw, Dararat Prayongkul, Prida Malasit, Panisadee Avirutnan, Sansanee Noisakran, Noppadon Nuntawong

**Affiliations:** † Spectroscopic and Sensing Devices Research Group, National Electronics and Computer Technology (NECTEC), National Science and Technology Development Agency (NSTDA), Pathum Thani 12120, Thailand; ‡ Molecular Biology of Dengue and Flaviviruses Research Team, Medical Molecular Biotechnology Research Group, National Center for Genetic Engineering and Biotechnology (BIOTEC), National Science and Technology Development Agency (NSTDA), Bangkok 10700, Thailand; § The Division of Dengue Hemorrhagic Fever Research (DHFR) and Siriraj Center of Research Excellence in Dengue and Emerging Pathogens, 65106 Faculty of Medicine Siriraj Hospital, Mahidol University, Bangkok 10700, Thailand

## Abstract

This study introduces a novel approach to dengue diagnostics by leveraging surface-enhanced Raman spectroscopy (SERS) coupled to machine learning. This method addresses the critical need for rapid and accurate identification of dengue virus (DENV) infection and prediction of the disease severity. For the first time, a commercialized SERS substrate is applied to analyze plasma samples from 60 pediatric patients, equally distributed among other febrile illnesses (OFI), dengue fever (DF), and dengue hemorrhagic fever (DHF) cases. This innovative application of SERS technology captures unique molecular signature characteristics of each disease state, offering a new paradigm in viral diagnostics. Our methodology employs various machine learning algorithms, notably linear discriminant analysis (LDA) and logistic regression, to classify the SERS spectral data. The models exhibited exceptional performance in distinguishing dengue from OFI, with both achieving an outstanding area under the curve (AUC) of 0.99. In the more complex task of discriminating between DF, DHF, and OFI, LDA demonstrated remarkable AUC values of 0.81, 0.90, and 0.99, respectively, while logistic regression slightly outperformed with AUC values of 0.82, 0.88, and 0.99. Even in the challenging differentiation of DF from DHF, the models achieved notable AUC values of 0.84 (LDA) and 0.79 (logistic regression). This pioneering SERS-based approach represents a significant advancement over existing dengue diagnostic methods, offering unparalleled speed and accuracy, particularly in resource-limited settings. By providing a new tool for early detection and classification of dengue severity, this innovative technique has the potential to improve patient outcomes and guide targeted therapeutic strategies in dengue management.

## Graphical abstract

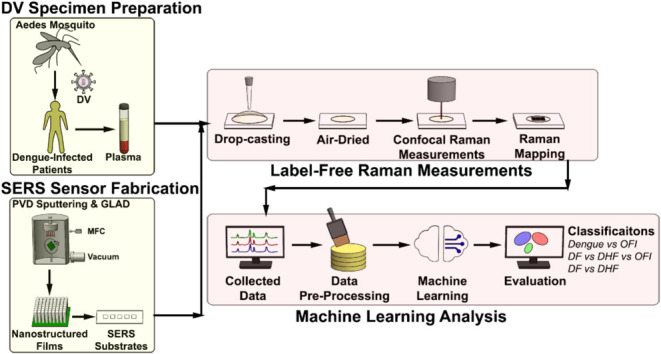

Dengue virus (DENV) infections pose a significant global health threat, with an estimated 390 million cases occurring annually, leading to substantial morbidity and mortality worldwide.^1^ This mosquito-borne disease disproportionately affects tropical and subtropical regions, where the most severely affected populations vary. For example, in Southeast Asia, dengue is a leading cause of hospitalization and death among children, whereas in areas like Brazil, adults are often more severely impacted.^2^ The incidence and geographic range of dengue are expanding due to factors such as climate change, urbanization, travel, and trade, which create more favorable conditions for mosquito vectors and viral transmission.^3^

Although most DENV infections are asymptomatic, approximately one-fourth of affected individuals manifest a range of clinical presentations from mild fever to severe, life-threatening complications. The World Health Organization (WHO) classified dengue into various categories to facilitate clinical management. While the WHO revised its dengue classification criteria in 2009 ^4^, the 1997 WHO criteria remain relevant for research purposes and provide a valuable framework for understanding the progression and severity of dengue illness. The criteria categorize the disease into dengue fever (DF) and dengue hemorrhagic fever (DHF). DHF represents the more severe form of dengue, characterized by plasma leakage, which can lead to shock (dengue shock syndrome, DSS) and potentially fatal complications.^5^

Currently, the diagnosis of dengue relies heavily on clinical manifestations, which are often indistinguishable from those of other febrile illnesses (OFI). Laboratory tests for viral products or specific antibody responses in blood specimens are essential for their confirmation. However, these tests have limitations. Rapid diagnostic tests for DENV nonstructural protein 1 (NS1) or anti-DENV antibodies can have varying sensitivity depending on the timing of sample collection.^6^ More reliable methods like enzyme-linked immunosorbent assay (ELISA) and reverse-transcription polymerase chain reaction (RT-PCR) require specialized laboratory equipment and expertise, making them time-consuming and costly.^7–9^ Furthermore, predicting which patients will develop severe dengue remains a significant challenge.^10,11^ A small percentage of DENV-infected individuals progress to severe disease around the time of defervescence, but the underlying mechanisms are not fully understood.^12^ The lack of prognostic tools and specific treatments underscores the urgent need for accurate and timely diagnosis, especially in resource-limited settings, where access to advanced laboratory testing is often limited.

Surface-enhanced Raman spectroscopy (SERS) has emerged as a promising technique for medical diagnostics due to its unique ability to provide label-free, highly sensitive, and specific molecular information.^13–16^ This methodology leverages the amplification of Raman scattering by nanostructured metal surfaces to detect spectral fingerprints of molecules, such as proteins and nucleic acids, in biological samples.^17,18^ SERS offers several advantages over conventional methods. Its rapid analysis capabilities can provide results within minutes, facilitating timely diagnosis and treatment decisions, which is crucial in managing DENV infections, especially in children, where disease progression can be rapid. Furthermore, SERS often requires minimal sample preparation, making it suitable for point-of-care testing in resource-limited settings. The technique’s intrinsic capability to identify subtle changes in molecular compositions makes it an attractive tool for disease diagnostics, including viral infections.^19–21^ Previous studies have demonstrated the potential of SERS in various applications for dengue diagnosis. For instance, researchers have combined SERS with lateral flow assays to detect dengue NS1 antigen in human sera^22^ and explored its use for noninvasive detection of DENV NS1 in saliva using a principal component analysis integrated with a support vector machine (PCA-SVM) model.^23^ Another study showcased the ability of a SERS platform, using a hand-held Raman spectrometer and PCA analysis, to differentiate DENV-infected patients from healthy individuals based on serum analysis.^24^

Building upon these advancements in SERS technology and our previous work developing a commercialized SERS substrate^25–27^ , this study investigates the potential of SERS coupled with machine learning algorithms to enhance the rapid and accurate diagnosis of dengue in children. Specifically, our research goal is to discriminate among DF, DHF, and other febrile illnesses (OFI) using plasma samples from pediatric patients. By analyzing the unique molecular signatures captured by SERS and supervised machine learning algorithms, we aim to develop a novel diagnostic tool with the potential to improve patient management and contribute to the control of dengue outbreaks.

## Experimental Section

### Plasma Samples from Pediatric Patients

Sixty plasma samples from pediatric patients (DF, DHF, and OFI patients, *n* = 20 in each group) were used in this study under approval by the Ethics Committee of Siriraj Institutional Review Board (Si 657/2023), the Siriraj Safety Risk Management Taskforce (SI 2023–065), and the Institution Biosafety Committee of the National Science and Technology Development Agency (IBC-0007–2566). The plasma samples were obtained from two dengue clinical cohorts at the Khon Kaen Hospital and Songkhla Hospital in Thailand. These samples were collected on the day prior to defervescence (namely, day −1). All dengue patients were confirmed for DENV infection by DENV RT-PCR, DENV NS1 ELISA, and DENV IgM/IgG capture ELISA, whereas patients with other febrile illnesses (OFI) tested negative for these assays. Differentiation between DF and DHF groups among dengue patients was performed according to the 1997 World Health Organization classification.^5^

### SERS Substrate Fabrication and Characterization

Ag substrates were prepared using a customized DC magnetron sputtering system with the glancing angle deposition (GLAD) technique.^28^ The detailed description of the SERS substrate fabrication is presented in the Supplementary Method S1.

### SERS Measurements

Raman measurements were performed by Raman spectroscopy (Invia Renishaw, UK) using an excitation laser source at 785 nm. The laser power was maintained at 20 mW. A 50× objective lens was used to focus the laser beam of approximately 1.2 μm in diameter onto a targeted spot. The grating was 1200 l/mm, and the exposure time was 10 s. Plasma samples (2 μL) were dispersed onto the SERS substrates and air-dried at room temperature for 15 min before Raman spectral analysis. Raman signals were collected in 49 different areas per 5 × 5 mm^2^ substrate. The Raman system was calibrated using the 520 cm^–1^ band of a bare silicon wafer before measurement.

### Data Preprocessing

The spectral data of plasma samples measured by SERS underwent a comprehensive preprocessing pipeline prior to machine learning (ML) analysis. Figure S1 illustrates the step-by-step procedures to prepare the raw data for subsequent ML classification of the disease groups (OFI, DF, and DHF) that was implemented using the Python programming software with essential libraries such as NumPy, SciPy, Rampy, and Sci-kit learn. Data preprocessing began with importing the raw data obtained from surface-mapped Raman spectroscopic measurements. Next, the acquisition position information was discarded, and only the raw spectral data were extracted to ensure data consistency and facilitate further analysis. Cubic spline interpolation was used to transform the original data into an equally spaced Raman shift frequency of 1 cm^–l^ between each data point due to its ability to effectively remove background noise without compromising the data integrity. To address anomalous spikes in the spectra, a cosmic ray removal technique was employed using the Whitaker-Hayes algorithm.^29^ This algorithm detects and removes anomalous spikes based on modified Z scores, ensuring a cleaner and more reliable data set for subsequent analysis. Outlier removal was also implemented to eliminate spectra that demonstrated significant deviations from the majority of the data. Principal component analysis (PCA) was employed to project the data set into a high-dimensional space, and the first 5 principal components were retained for outlier removal. Spectra outside the 95% confidence interval of the Mahalanobis distance were identified as outliers and consequently removed from the data set. Baseline correction was applied to further refine the data. A polynomial function of degree 5 was fitted to an individual spectrum, and the resulting curve was subtracted from the corresponding data points, effectively removing baseline variations. The final preprocessed data, which were devoid of background noise, anomalous spikes, outliers, and baseline variations, were fed into the ML algorithms for classification. These algorithms employ various techniques to learn from the data and classify dengue (DF and DHF) and OFI groups based on the distinctive SERS spectra of plasma samples. The present study followed this comprehensive and systematic flowchart (Figure S1) to ensure the reliability and accuracy of the group classification process.

### Data and Machine Learning Analyses

Before applying machine learning algorithms, we first conducted PCA and LDA score plots to visualize data clusters and overlaps from the preprocessed SERS spectra. This analysis helped us understand the underlying structure of the data based on the maximized variance or covariance. To accurately classify plasma samples into their respective disease categories (DF, DHF, and OFI), our experimentation was structured into three distinct scenarios: (a) binary classification between dengue and OFI, where dengue represents the combined DF and DHF groups; (b) ternary classification among DF, DHF, and OFI; and (c) binary classification between DF and DHF. For each scenario, the labeled spectra were aggregated into a training set. A total of 49 individual SERS spectra were collected for each patient sample. To maximize the information available for model training and to account for intrasample variability, all viable spectra (approximately 2900 in total after outlier removal) were used to train the machine learning models. Performance, however, was evaluated at the patient level. For classification, predictions were made on all 49 spectra from a given sample, and a final diagnosis was determined via a majority-vote system. For example, in the binary DHF vs DF classification, a sample was assigned the “DHF” label if at least 25 of its spectra were classified as DHF. This patient-centric evaluation framework ensures that the reported metrics in the confusion matrices (*n* = 60 or *n* = 40) reflect clinically relevant performance.

A variety of machine learning algorithms toward classifications of unique spectral profiles extracted from the SERS measurements have been used in this study. These included linear discriminant analysis (LDA), logistic regression (LR), principal component analysis-based LDA (PCA-LDA), multilayer perceptron (MLP), support vector machine (SVM), decision tree (DT), and random forest (RF). Each model offers a unique algorithmic approach, influencing its effectiveness in classification tasks. The choice of the most suitable model depended on the specific nature of the classification task, emphasizing the importance of nuanced distinctions between disease groups or broader categorizations.

To identify the best ML models, leave-one-out cross-validation (LOOCV) was performed on the 60-sample set, ensuring robust model selection by iteratively training the models on all samples except one and validating on the excluded sample. Additionally, we conducted a receiver operating characteristic (ROC) analysis of the two best models selected from the LOOCV stage. This essential step involved calculating the area under the curve (AUC) to comprehensively evaluate the models’ discriminatory power and sensitivity across different thresholds. The ROC analysis provided crucial insights into each model’s performance, particularly in distinguishing between dengue (DF and DHF) and OFI groups. This strategic examination was imperative to validate the efficacy of the models in capturing true positive rates while minimizing false positives across a range of classification thresholds. Moreover, to gain further insights into the 3-group scenario (DF, DHF, and OFI), we analyzed the inverse spectra from group centroids in the PCA scatter plots. This involved performing the Moore-Penrose pseudoinverse of the reduced matrices generated by the PCA models. This analysis aimed to explore the potential explainability of the machine learning model for these data sets, thereby enhancing the interpretability and understanding of the discriminatory features present in the data.

## Results and Discussion

### Clinical and Demographic Profiles of Pediatric Patients

This study analyzed 60 plasma samples from pediatric patients aged 5 to 14 years. The patients were divided into three groups: DF, DHF, and OFI. The DF and DHF groups each consisted of 20 patients (*n* = 5 for each DENV serotype: DENV-1, DENV-2, DENV-3, and DENV-4). The OFI group consisted of 20 patients with febrile illnesses unrelated to dengue. All plasma samples were collected on the day before defervescence (day −1), corresponding to 4 to 10 days after fever onset, and stored appropriately before subsequent SERS and machine learning analysis. Demographic and clinical laboratory data for each patient are provided in supplementary Tables S1–S3. Figure 1 summarizes the key clinical and demographic characteristics of the three patient groups. As expected, both DF and DHF patients exhibited significantly higher hematocrit levels and lower platelet and white blood cell counts compared to those of OFI patients (Figure 1b). These findings are consistent with the typical hematological manifestations of DENV infection. DHF patients exhibited significantly elevated levels of liver enzymes, aspartate transaminase (AST) and alanine transaminase (ALT), compared to OFI patients (Figure 1b), indicating potential liver involvement in severe dengue cases. Albumin levels did not differ among the three patient groups. Notably, DENV NS1 levels did not differ significantly between DF and DHF patients (Figure 1b).

**Figure 1:**
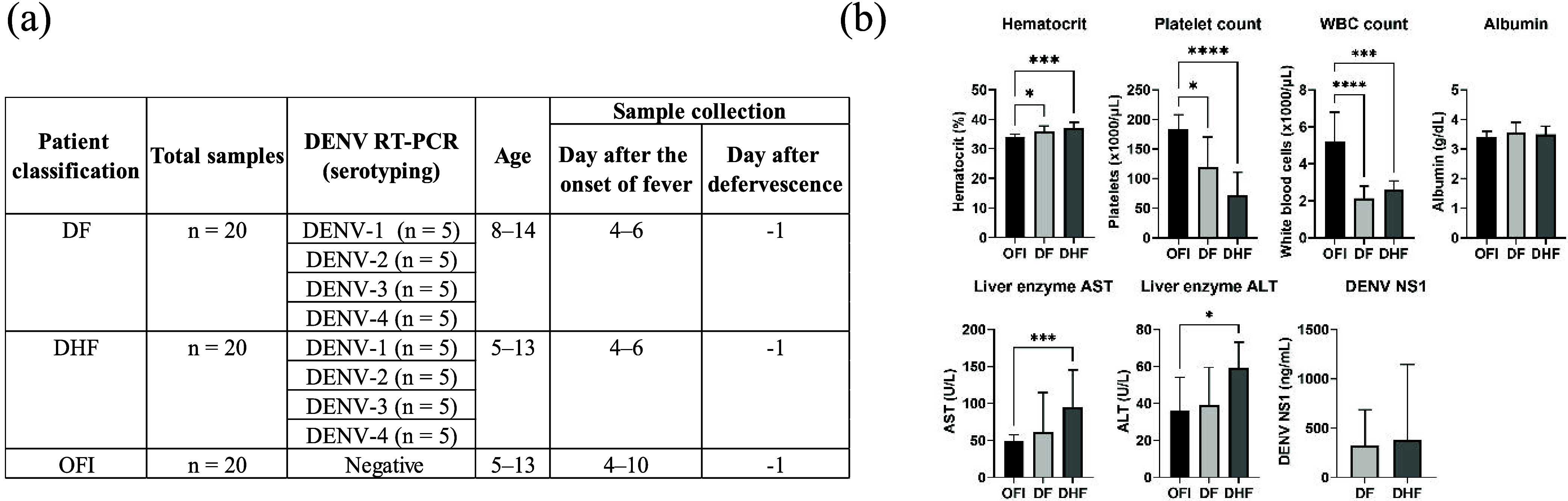
Demographic and clinical profiles of OFI, DF, and DHF patients. (a) Sample distribution across disease classifications and infecting DENV serotypes. Samples were collected on the day before defervescence (day −1), which corresponds to 4 to 10 days after fever onset. (b) Comparison of hematocrit, platelet count, white blood cell count, albumin, liver enzymes (AST and ALT), and DENV NS1 levels between patient groups (median with interquartile range). Significant differences between groups were analyzed using the Kruskal–Wallis test with Dunn’s multiple comparisons (*, *P* < 0.05; ***, *P* < 0.001; ****, *P* < 0.0001).

### Characterization of SERS Chips

To ensure the reliability and sensitivity of our SERS-based dengue diagnostic approach, we first characterized the physical properties of the SERS chips. Figure S2 shows SEM images of (a) top and (b) cross-sectional views of Ag nanorods grown on a Si substrate. Vertically aligned Ag nanorods with varying diameters were observed on the silicon surface. As shown in Figure S2a, the average diameter of these Ag nanorods was 118.99 nm with a standard deviation of 19.81 nm, and the density was approximately 72 nanorods/μm^2^. The cross-sectional SEM view in Figure S2b indicates a tightly packed arrangement of the Ag nanorods with an axis length of approximately 324.56 ± 5.80 nm. These characteristics are consistent with the expected morphology for SERS substrates fabricated using the GLAD technique with substrate rotation. Our previous studies have demonstrated that glancing angles (θ) greater than 70° with an optimum rotation speed during fabrication can generate ordered columnar nanostructures on the SERS chips.^30^ While the rotation speed and self-shadowing effect can influence the size variation of the nanorods^31^, the observed variation in our chips is within an acceptable range for effective SERS performance.

### Data Analysis and Machine Learning

To analyze the biochemical composition of the plasma samples, we performed SERS measurements, obtaining Raman spectra that provide unique molecular signature characteristics of each disease state. This spectral data formed the foundation data set for subsequent analysis using supervised machine learning algorithms to identify key features associated with DENV infection and disease severity.

Figure 2a shows the averaged preprocessed spectra of each group (OFI, DF, and DHF) with stacking offsets. Additional representations of all obtained Raman spectra are shown in Figure S3. Despite their overall similarity, subtle differences in some vibrational bands can be observed between the three groups (Figure 2a and S3). The resemblance between the spectra makes it challenging to distinguish distinct variations based on conventional Raman band assignments.

**Figure 2:**
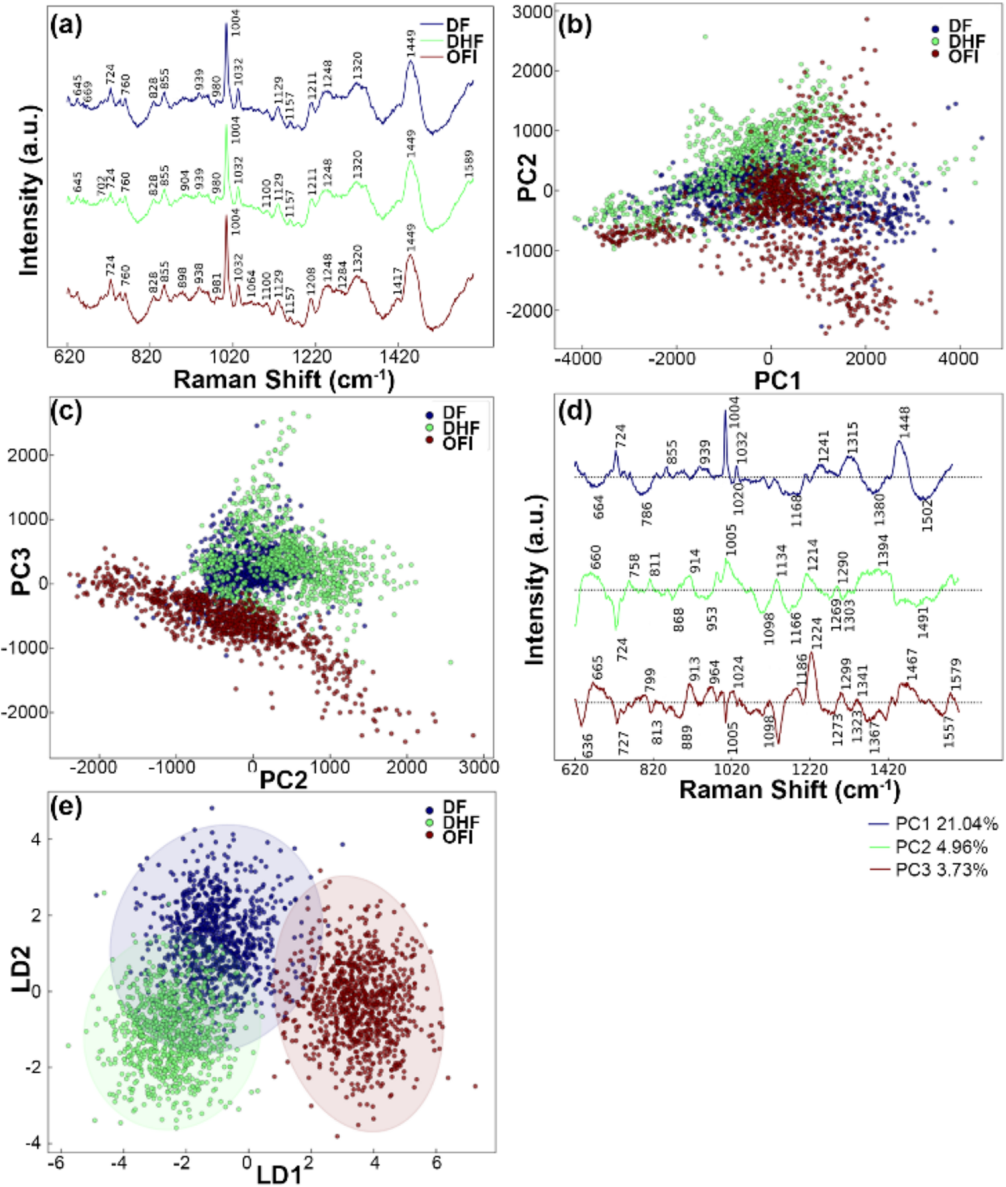
Analysis of Raman spectra from OFI, DF, and DHF patients. (a) Average SERS spectra. (b) PCA score plot (PC1 vs PC2) for dimensional reduction and visualization. (c) PCA score plot (PC2 vs PC3) highlighting the separation between OFI and dengue groups (DF and DHF). (d) PCA loading plots for PC1, PC2, and PC3 with their respective explained variance ratios. (e) LDA score plot (LD1 vs LD2) for classification, showing separation between groups. OFI: other febrile illnesses; DF: dengue fever; DHF: dengue hemorrhagic fever.

To visualize the relationship between the different sample groups based on their Raman spectra, we performed principal component analysis (PCA). Figure 2b illustrates a PCA score plot graphically representing the distribution of the data along the first two principal components (PC1 and PC2). The plot reveals significant overlap among the three groups, indicating a lack of clear discrimination based on these principal components. Figure 2c presents an additional PCA score plot between PC2 and PC3, emphasizing a notable separation of the OFI group from the DF and DHF groups. However, the DF and DHF clusters still exhibited significant overlap, making it challenging to distinguish between these two categories solely on the basis of these principal components. The loading plots for PC1, PC2, and PC3, as shown in Figure 2d, reveal which wavenumbers contribute most significantly to the variance captured in each principal component. The PC2 and PC3 loadings, in particular, highlight the spectral differences that drive the separation between the OFI and dengue patients. These loading patterns correspond to molecular signatures associated with dengue infection, providing insights into the biochemical basis of the observed group clustering.

While PCA is useful for dimensionality reduction and visualization of overall data patterns, it may not optimally separate distinct classes. Therefore, we employed linear discrimination analysis (LDA), which is specifically designed to maximize the separation between classes for improved classification. Transitioning from PCA to LDA allows us to examine the data much deeper and potentially uncover subtler patterns that might not be apparent with PCA alone. Figure 2e illustrates the LDA score plot, representing LD1 and LD2 with 95% confidence ellipses delineating each group. This plot demonstrates a clear separation between the OFI and DHF groups. However, the DF group shows a slight overlap with the OFI and DHF groups, implying a complex discriminatory pattern for distinguishing the DF group from the other groups.

To further investigate the spectral differences between the patient groups, we analyzed the inverse spectra of the PCA calculated based on the center data point of each group (Figure 3). Using the inversed PCA spectra from the cluster center of each group offers several advantages over using the averaged spectra. It enhances discriminability by capturing the most representative spectral features of each group while reducing noise and variability inherent in individual spectra. Peaks were annotated based on specific criteria to ensure that only significant spectral features were identified. A peak was considered significant if it met the following criteria: (1) a signal-to-noise (S/N) ratio greater than 5; (2) a prominence value exceeding 3; and (3) a width falling within the range of 3 to 7 data points. This approach ensures that only relevant peaks with sufficient prominence and width are annotated, enhancing the accuracy and reliability of the peak identification in the SERS spectra.

**Figure 3:**
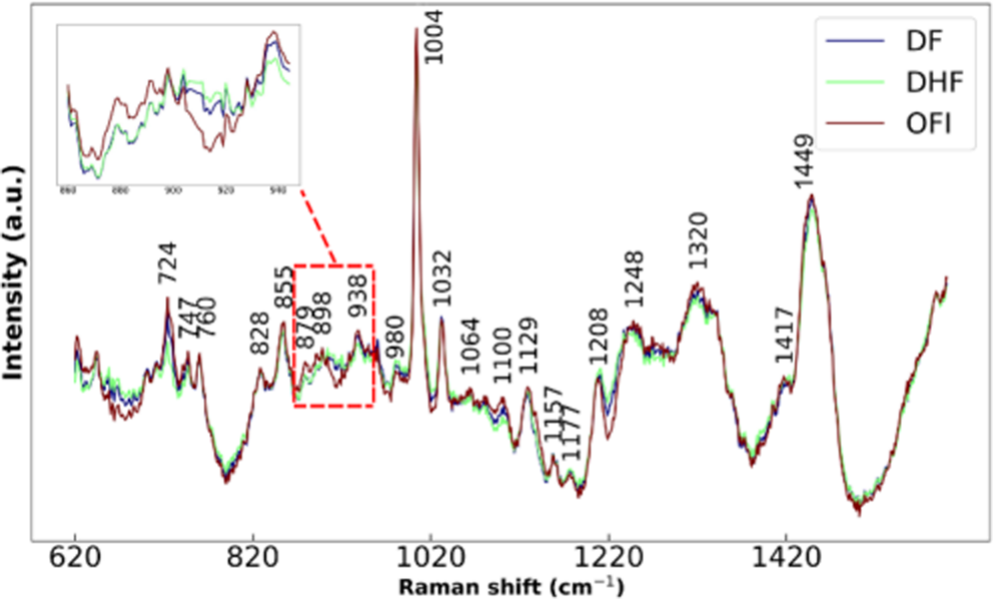
Inversed PCA spectra calculated based on the centered data point of each group. This analysis was performed to ensure that the original principal components were not biased toward features with larger means, thus providing a more accurate representation of the data’s true structure.

To identify the specific molecular changes associated with DENV infection, we analyzed the Raman spectra obtained from the SERS measurements. The Raman shifts observed in the spectra correspond to specific molecular vibrations, providing insights into the biochemical composition of the plasma samples. Table S4 presents the assignment of major peaks for all samples based on the obtained Raman shifts. Some of the identified Raman peaks in our study showed Raman shifts relatively similar to those reported previously using human sera infected with DENV.^32,33^

A detailed analysis of the inverse PCA spectra (Figure 3) revealed significant differences between the DENV-infected groups (DF and DHF) and the OFI group. Notably, the DF and DHF groups exhibited shifts to higher wavenumbers in the broad SERS signals around 870 and 930 cm^–1^ compared to the OFI group (Inset plot of Figure 3). The spectral shift at 879 cm^–1^, attributed to the antisymmetric stretch vibration of choline and phospholipids (Table S4), suggests an alteration in cellular membrane components, which are crucial for DENV entry and replication.^34^ Furthermore, the bands at 898 cm^–1^ (in-plane bending of deoxyribose), 904 cm^–1^ (C–O–C skeleton mode of glucose), and 938 cm^–1^ (C–C stretching of proteins) (Table S4) may reflect metabolic changes and host responses to DENV infection.^35^ Additionally, the band at 1208 cm^–1^, elevated in DENV-infected samples, is likely involved in the amide III−β conformation of proteins (Table S4), with contributions from the C6H5–C stretching vibrations of tryptophan and phenylalanine. This observation aligns with previous reports of elevated serum levels of phenylalanine in DENV infections.^36^ Importantly, these reconstructed spectral features are consistent with the PCA loading patterns. For example, the PC3 loading plot (Figure 2d) shows prominent contributions at 889 cm^–1^ (negative loading) and 913 cm^–1^ (positive loading), aligning with the differences in the 870–930 cm^–1^ region observed in the inverse spectra. This correspondence supports the validity of both PCA decomposition and the spectral differences reconstructed from group-level centroids.

These findings highlight the intricacies of SERS spectral analysis in differentiating DF and DHF, underscoring the potential of the proposed method to capture subtle molecular variations indicative of dengue-related illnesses even in the presence of spectral overlaps. This complexity further underscores the need for advanced analytical techniques, such as machine learning, to effectively classify and diagnose DENV infections based on SERS data.

To evaluate the performance of our machine learning approach in different diagnostic contexts, we investigated three scenarios: (1) binary classification of dengue versus OFI, (2) multiclass classification of DF, DHF, and OFI, and (3) binary classification of DF versus DHF. For the first scenario, we performed binary classification to differentiate between the dengue group (including DF and DHF) and the OFI group. Table 1a presents the performance metrics of various machine learning models using leave-one-out cross-validation (LOOCV) on a data set of 60 samples. We employed LOOCV to assess the generalizability of the models, providing robust performance assessment and insights into potential overfitting by testing the model on unseen data in each iteration, especially given the limited sample size.

**Table 1:**
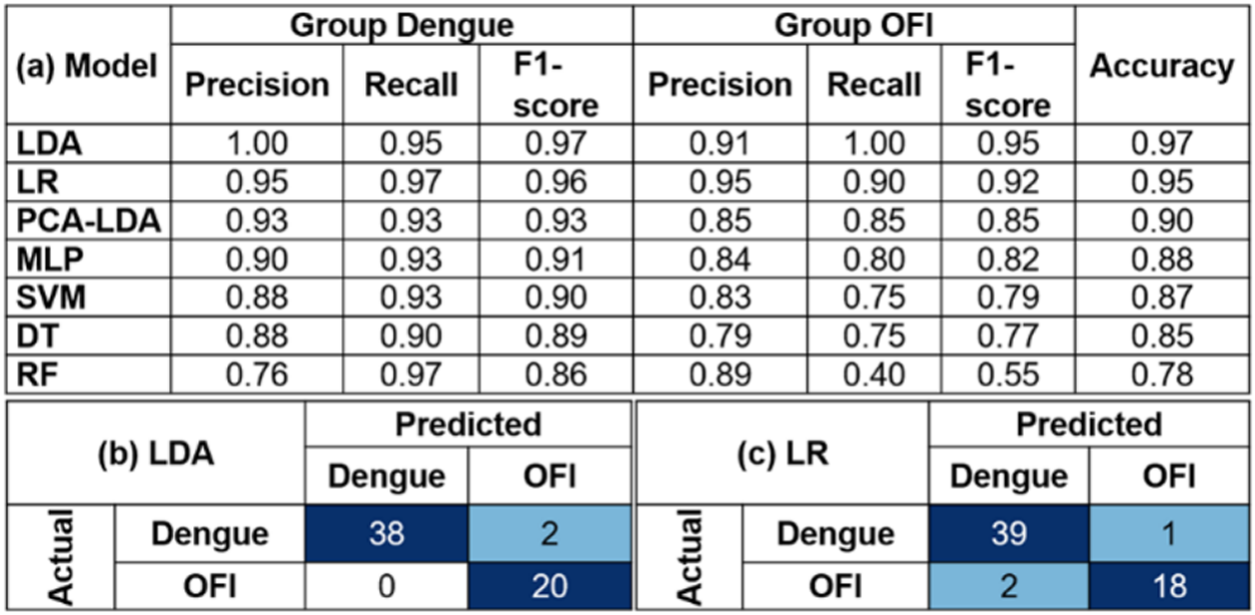
Performance of Machine Learning Models in Differentiating between Dengue (Including DF and DHF) and OFI Groups Using a Data Set of 60 Plasma Samples^a^

a Note: (a) Performance metrics of machine learning models, showing precision, recall, and F1-score for each model in classifying dengue and OFI, using leave-one-out cross-validation (LOOCV) to assess model generalizability. Confusion matrices showing the number of true positive, true negative, false positive, and false negative predictions for the top-performing models: (b) LDA and (c) logistic regression. Abbreviations for models: LDA (linear discriminant analysis); LR (logistic regression); PCA (principal component analysis); MLP (multilayer perceptron); SVM (support vector machine); DT (decision tree); and RF (random forest).

As shown in Table 1a, LDA, logistic regression, and PCA-LDA demonstrate high precision, recall, and F1-scores for both dengue and OFI, highlighting their robustness in classification tasks. High precision is particularly crucial in a diagnostic setting, as it minimizes false positive results, ensuring that individuals identified as having dengue are truly infected. LDA stands out with perfect precision (1.00) in the dengue group, indicating that all instances it predicted as dengue were indeed correct. This high precision is coupled with a high recall (0.95) and F1-score (0.97). Logistic regression follows closely, showcasing a balanced performance with slightly lower precision but impressive recall and F1-score. PCA-LDA exhibits consistent accuracy but with a slightly lower F1-score for OFI. Additionally, the MLP, SVM, and decision tree models also display competitive performance, albeit slightly lower than the top-performing models. Random forest demonstrates high recall for dengue but significantly lower recall and F1-score for OFI, indicating potential overfitting or bias toward dengue.

Finally, Table 1b,c present the confusion matrices of the top two models: LDA and logistic regression, respectively. The confusion matrices visually confirm the high performance of LDA and logistic regression, showing minimal misclassifications. LDA demonstrates a nearly perfect classification, with only two dengue samples misclassified as OFI. Logistic regression exhibits a similar trend, misclassifying two OFI samples as dengue and one dengue sample as OFI.

Next, we evaluated the performance of the machine learning models in a ternary classification scenario, aiming to differentiate between DF, DHF, and OFI. Ternary classification presents a greater challenge due to the increased complexity of distinguishing between three distinct classes, particularly with the overlapping features of DF and DHF. Table 2a shows the performance metrics of the different machine learning models in these ternary classification tasks. Logistic regression excelled in identifying OFI but struggled with differentiating DF and DHF. LDA showed a more balanced performance across all groups, effectively distinguishing DHF from OFI, although with some difficulty in identifying DF. This suggests that LDA may be better suited for handling the complexities of ternary classification in this context. Other models, such as PCA-LDA and MLP, showed moderate performances, while random forest, decision tree, and SVM exhibited more variable performance. SVM revealed the lowest overall performance, particularly struggling with precision and recall for the DF and DHF groups.

**Table 2:**
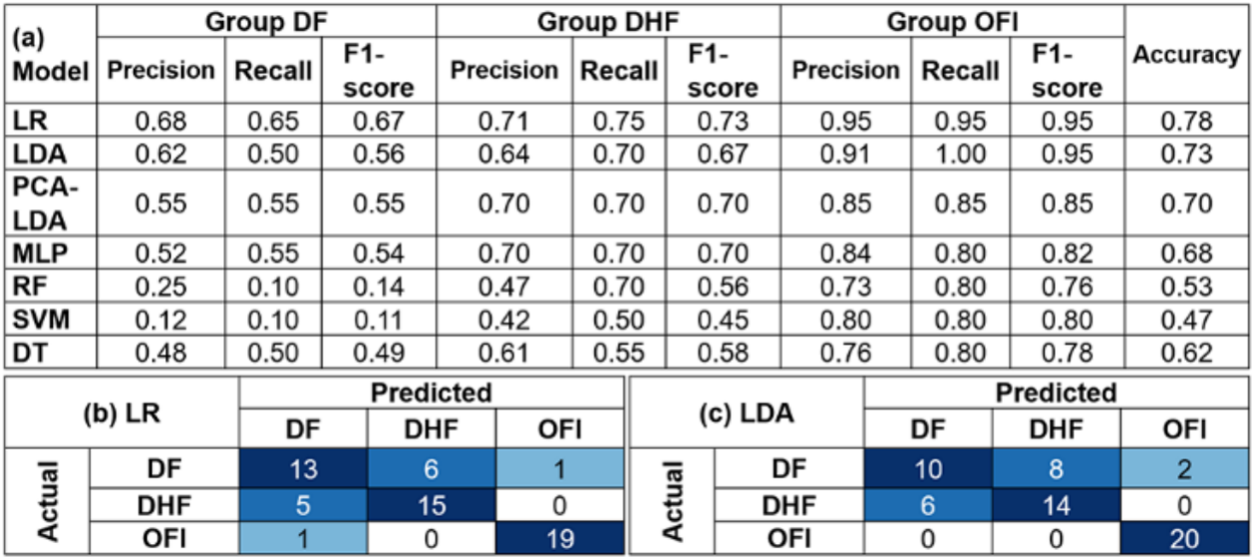
Performance of Machine Learning Models in the Three-Group Classification Scenario (DF, DHF, and OFI) Using a Data Set of 60 Plasma Samples^a^

a Note: (a) Performance metrics of machine learning models, showing precision, recall, and F1-score for each model in classifying DF, DHF, and OFI, using LOOCV to assess model generalizability. Confusion matrices showing the number of true positive, true negative, false positive, and false negative predictions for the top-performing models: (b) logistic regression and (c) LDA models.

The confusion matrices (Table 2b,c) illustrate the misclassification patterns of the models. Logistic regression consistently misclassified some DF instances as DHF, while LDA showed a more balanced misclassification pattern, primarily between DF and DHF. These analyses emphasize the challenging nature of distinguishing between DF and DHF due to their overlapping features.

These findings highlight the need for further model refinement and feature engineering to enhance the discrimination between DF and DHF, ultimately improving the accuracy and reliability of dengue diagnosis. Refining these models may yield more accurate and reliable classification outcomes, which are crucial for precise dengue diagnosis and subsequent medical interventions.

Finally, we evaluated the performance of the machine learning models in a binary classification scenario, specifically focusing on differentiating between DF and DHF. Accurate differentiation between DF and DHF is crucial for guiding clinical management and ensuring timely interventions to prevent severe complications associated with DHF. Table 3a depicts the evaluation of machine learning models in this binary classification task. Logistic regression, PCA-LDA, and MLP demonstrated consistent performance in classifying both DF and DHF, with balanced precision, recall, and F1-scores averaging around 0.70 for both groups. Despite their moderate accuracy, these models consistently demonstrate their competence in distinguishing between the two dengue patient groups. In contrast, the random forest, SVM, and decision tree models exhibit varying performances. While the decision tree shows equivalent precision, recall, and F1-scores for both groups, random forest and SVM display comparatively lower performance across all evaluation metrics. Notably, LDA emerges as a standout among the evaluated models. It demonstrates superior precision, recall, and F1-scores for both the DF and DHF groups, averaging around 0.78. LDA showcases a significantly balanced classification between the two dengue patient groups, displaying the highest overall performance in this binary classification task.

**Table 3:**
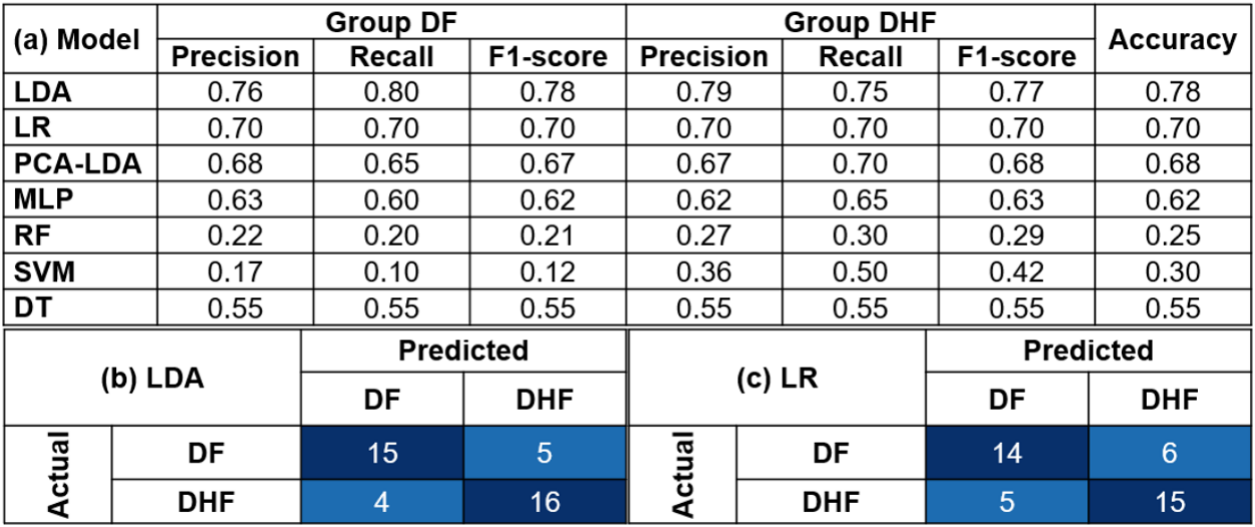
Performance of Machine Learning Models in Differentiating between DF and DHF Using a Data Set of 40 Plasma Samples^a^

a Note: (a) Performance metrics of machine learning models, showing precision, recall, and F1-score for each model in classifying DF and DHF, using LOOCV to assess model generalizability. Confusion matrices showing the number of true positives, true negatives, false positives, and false negative predictions for the top-performing models: (b) LDA and (c) logistic regression.

The confusion matrices, as shown in Table 3b,c, illustrate the strong performance of LDA and logistic regression in differentiating DF and DHF. The LDA model correctly classified 15 instances of DF and 16 instances of DHF, while logistic regression correctly classified 14 instances of DF and 15 instances of DHF. These results further support the high accuracy of these models in distinguishing between the two dengue patient groups.

Receiver operating characteristic (ROC) curves provide a comprehensive visualization of a classification model’s performance across different thresholds. They plot the true positive rate against the false positive rate, allowing the assessment of the model’s ability to discriminate between classes. Figure 4 shows the ROC curves for LDA and logistic regression in each of the three classification scenarios. These curves provide an in-depth depiction of the discriminating capabilities of the two best models selected from the LOOCV stage.

**Figure 4:**
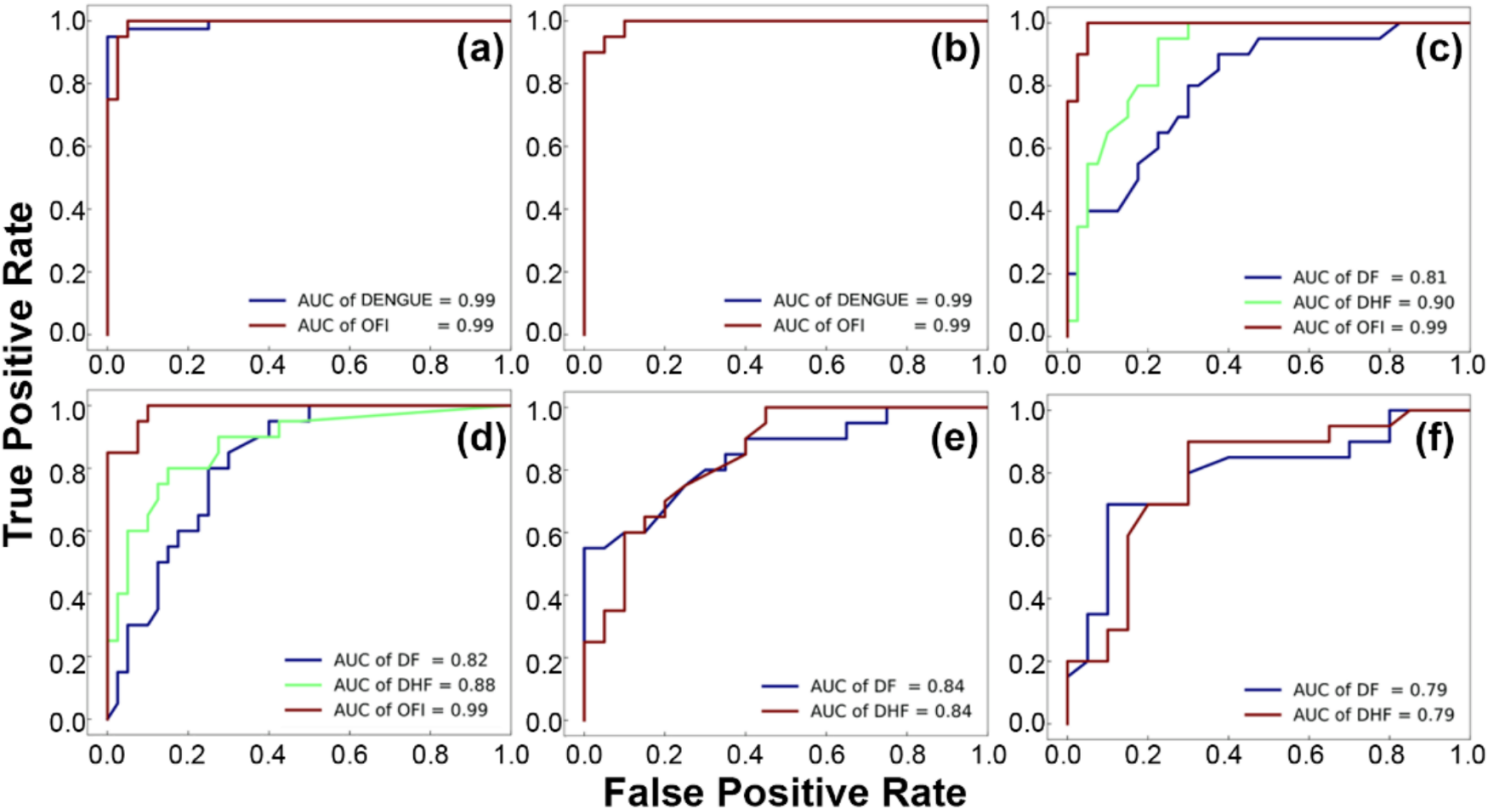
ROC curves illustrating the discriminative capabilities of LDA and logistic regression across various dengue classification scenarios. (a and b) Scenario 1: dengue (including DF and DHF) versus OFI (LDA, then logistic regression). (c and d) Scenario 2: DF versus DHF versus OFI (LDA, then logistic regression). (e) and (f) Scenario 3: DF versus DHF (LDA, then logistic regression).

In Scenario 1, which involved discriminating between dengue and OFI, both the LDA and logistic regression models exhibited exceptional performance, displaying high AUC values of 0.99 for both dengue and OFI. These results indicate the robustness and reliability of both models in distinguishing between the two groups in this binary classification task.

In Scenario 2, where the classification task extended to include DF, DHF, and OFI, the LDA model displayed good discrimination with AUC values of 0.81 for DF, 0.90 for DHF, and 0.99 for OFI. In comparison, the logistic regression model demonstrated slightly improved performance with AUC values of 0.82 for DF, 0.88 for DHF, and 0.99 for OFI. These results reveal varying degrees of effectiveness in distinguishing between the three groups, with both models showing particularly strong discrimination for OFI but slightly less for DF and DHF.

In Scenario 3, targeting the distinction between DF and DHF, both the LDA and logistic regression models displayed varying performance. The LDA model exhibited an AUC value of 0.84 for both the DF and DHF groups. This finding indicates a comparable ability of the LDA model to differentiate between DF and DHF, showcasing a moderate but consistent discriminative capability for these two dengue patient groups. In contrast, the logistic regression model showcased slightly lower performance with AUC values of 0.79 for both DF and DHF. Although the logistic regression model still demonstrated reasonable discriminative ability between DF and DHF, its performance was slightly less optimal compared to the LDA model in this specific scenario.

Notably, both models consistently demonstrated high AUC values in distinguishing dengue (DF and DHF) from the OFI across all scenarios, highlighting the potential of our SERS-based approach for accurate dengue diagnosis. In this context, SERS offers a unique advantage in its ability to identify subtle molecular differences between the two closely related dengue subgroups (DF and DHF) with different severities and OFI patients. Its high sensitivity to molecular alterations presents a novel approach for precise dengue disease classification, complementing and enhancing existing diagnostic methodologies.

Although the discriminative performance between DF, DHF, and OFI was slightly lower, these models outperformed existing methods reliant on symptom observation and laboratory tests alone. The ability of these models to differentiate DF from DHF, even with moderate AUC values, holds promise for improved patient monitoring and risk stratification, potentially enabling earlier interventions to prevent severe complications. In comparison to current methods, which often lack the necessary specificity for timely DHF diagnosis, the proposed SERS-based approach offers a more refined and potentially earlier identification of the DHF onset.

Further investigation would be needed to improve the diagnostic and prognostic accuracy of dengue diseases with different severities using a large number of samples at varying time points after fever onset. Altogether, the present study indicates significant advancements in developing a new research tool for diagnosing dengue and predicting disease severity.

## Conclusions

This study demonstrates the feasibility of a novel SERS-based approach coupled with machine learning for the diagnosis of DENV infection in children. Our findings show that LDA and logistic regression models can effectively differentiate between dengue and other febrile illnesses based on SERS spectral data. This approach offers significant advancement over current diagnostic methods, which often rely on symptom observation and laboratory tests that may lack sensitivity and specificity, particularly in the early stages of an infection. Our SERS-based method achieved exceptional accuracy in differentiating dengue from other febrile illnesses with AUC values of 0.99. While differentiating between DF and DHF (AUC values ranging from 0.79 to 0.84) remains challenging, our results indicate the potential to significantly improve patient management, enabling timely interventions and reducing the risk of severe complications and mortality. The established SERS technique offers significant advantages for dengue diagnosis including high sensitivity, rapid results, and multiplexed detection capability. Its minimal sample preparation and adaptability for point-of-care use make it valuable in resource-limited settings. Further research is needed to address challenges such as the standardization of SERS substrates and the optimization of protocols for clinical use. Further studies with larger cohorts and longitudinal data will be crucial for validating the diagnostic and prognostic accuracy of this approach.

## Supplementary Material



## Notes

Methods for SERS substrate fabrication and characterization; preprocessing procedure of the Raman spectral data obtained from the SERS sensors for the classification of sample groups; SEM images of (a) top and (b) cross-sectional view of Ag nanostructures fabricated by GLAD sputtering to be utilized as the SERS sensors; Raman spectra from three different sample groups (OFI, DF, and DHF); demographic and clinical laboratory data of OFI patients; demographic and clinical laboratory data of DF patients; demographic and clinical laboratory data of DHF patients; and Raman band assignments for SERS spectra of plasma samples (PDF)
