# Supplementary material for: Discrimination of Dengue Diseases in Children Using Surface-Enhanced Raman Spectroscopy Coupled with Machine Learning Approaches
Source: Anal Chem. 2025 Jul 7;97(28):15122–31. doi: 10.1021/acs.analchem.5c01182 (PMC12291032; doi:10.1021/acs.analchem.5c01182)
Supplement: Supplementary file 1 [file ac5c01182_si_001.pdf]

Discrimination of Dengue Diseases in Children Using Surface-Enhanced Raman Spectroscopy Coupled with Machine Learning Approaches

Uraiwan Waiwijit,[†,||] Pitak Eiamchai,[†,||] Saksorn Limwichean,[†] Mati Horprathum,[†] Tanapan Prommool,[‡] Chunya Puttikhunt,[‡] Adisak Songjaeng,[§] Nuttapong Kaewjiw,[§] Dararat Prayongkul,[§] Prida Malasit,[§] Panisadee Avirutnan,[*,§] Sansanee Noisakran,[*,‡] and Noppadon Nuntawong[*,†]

[†] Spectroscopic and Sensing Devices Research Group, National Electronics and Computer Technology (NECTEC), National Science and Technology Development Agency (NSTDA), Pathum Thani 12120, Thailand

[‡] Molecular Biology of Dengue and Flaviviruses Research Team, Medical Molecular Biotechnology Research Group, National Center for Genetic Engineering and Biotechnology (BIOTEC), National Science and Technology Development Agency (NSTDA), Bangkok 10700, Thailand

[§] The Division of Dengue Hemorrhagic Fever Research (DHFR) and Siriraj Center of Research Excellence in Dengue and Emerging Pathogens, Faculty of Medicine Siriraj Hospital, Mahidol University, Bangkok 10700, Thailand

[||] These authors contribute equally to this work.

***Corresponding authors:**

Dr. Noppadon Nuntawong, National Electronics and Computer Technology (NECTEC), National Science and Technology Development Agency (NSTDA), Pathum Thani 12120, Thailand (E-mail: noppadon.nuntawong@nectec.or.th);

Dr. Sansanee Noisakran, National Center for Genetic Engineering and Biotechnology (BIOTEC), National Science and Technology Development Agency (NSTDA), Bangkok 10700, Thailand (E-mail: sansanee@biotec.or.th or snoisakran@yahoo.com);

Dr. Panisadee Avirutnan, Faculty of Medicine Siriraj Hospital, Mahidol University, Bangkok 10700, Thailand (E-mail: panisadee.avi@mahidol.edu).

Method S1. SERS Substrate Fabrication and Characterization. Ag substrates were prepared using a customized DC magnetron sputtering system with glancing angle deposition (GLAD) technique. A 3-inch Ag sputtering target was used for the deposition of the Ag nanostructures on a silicon (Si) base substrate of size approximately 5 × 5 mm2. Sputtering was carried out for 30 minutes under an operating pressure of 5.8 × 10−3 mbar with a constant flow of 28 SCCM Argon gas in the system. The voltage and current supply were kept constant at 300 V and 0.33 A, respectively. The Si substrate was rotated at 10 rpm during the sputtering process with an angle of 86 degrees to fabricate rod- shaped Ag nanostructures. Film morphology was then examined using a Hitachi SU8030 scanning electron microscope (SEM) operated at 10 kV accelerating voltage and 8.0 mm working distance equipped with energy-dispersive X-ray spectroscopy (EDS). SEM images were recorded at 50,000× magnification.

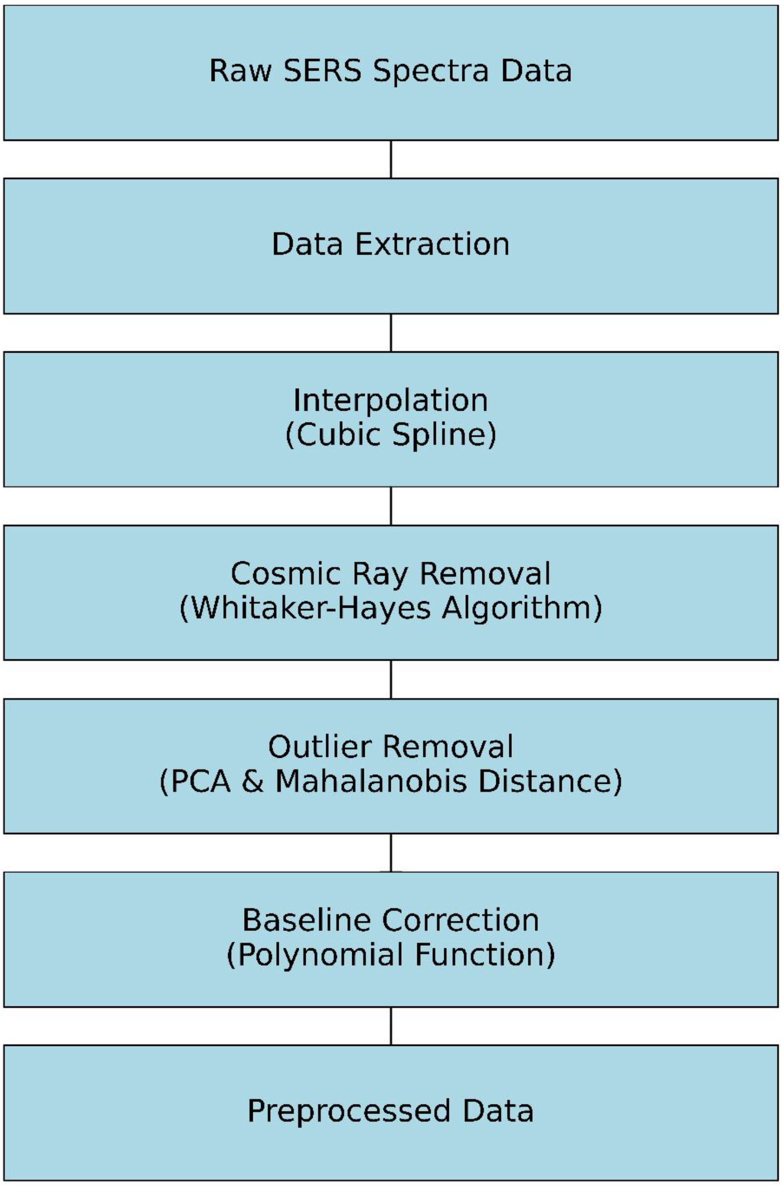

Figure S1. The preprocessing procedure of the Raman spectral data obtained from the SERS sensors for the classification of sample groups.

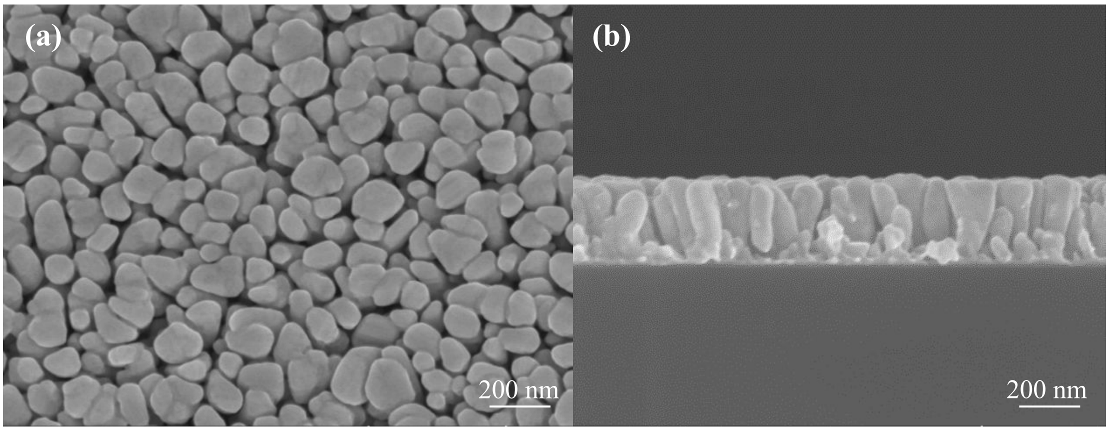

Figure S2. SEM images of (a) top and (b) cross-sectional view of Ag nanostructures fabricated by GLAD sputtering to be utilized as the SERS sensors.

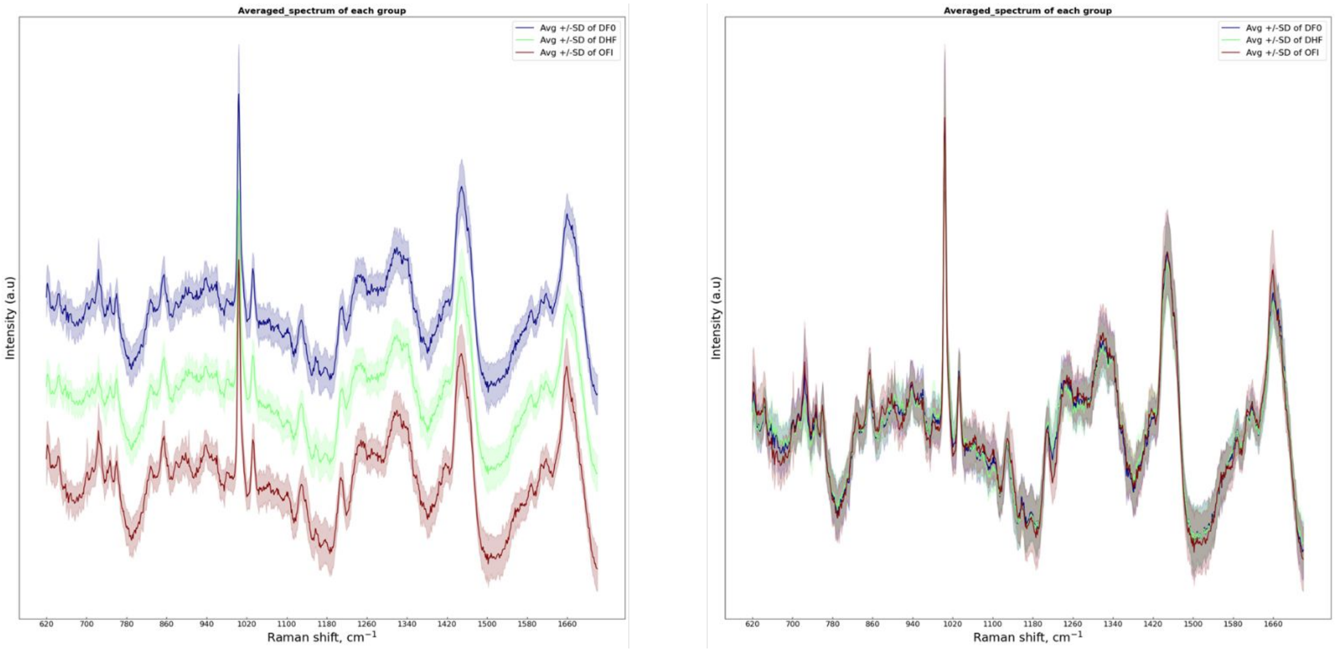

Figure S3. The Raman spectra from three different sample groups (OFI, DF, and DHF). The solid lines and shaded regions indicate the mean and standard deviations of spectra, respectively. (a) The stacked plot and (b) the overlay plot of the three groups.

Table S1. Demographic and clinical laboratory data of OFI patients.

Patient ID	Gender	Age	Day of sample collection		DENV RT-PCR (serotyping)	Hematocrit (%)	Platelets (x1,000/ml)	White blood cell count (x1,000 cells/ml)	Aspartate transaminase (AST, U/L)	Alanine transaminase (ALT, U/L)	Albumin (g/dl)	Classicification
			Based on fever onset	Based on defervescence								
A1	Female	9	4	-1	Negative	33	247	6.8	28	26	3.6	OFI
A2	Female	12	8	-1	Negative	29	215	5.3	155	87	3.1	OFI
A3	Female	10	5	-1	Negative	39	140	3	30	36	3.7	OFI
A4	Male	12	6	-1	Negative	34	101	1.7	84	54	2.9	OFI
A5	Female	8	8	-1	Negative	29	197	4.1	84	110	3.5	OFI
C1	Male	7	6	-1	Negative	NA	NA	NA	NA	NA	NA	OFI
C2	Female	8	9	-1	Negative	33	191	5.2	54	51	3.2	OFI
C3	Female	8	6	-1	Negative	35	117	4.6	50	64	3.5	OFI
C4	Female	10	7	-1	Negative	38	161	3.3	51	32	4	OFI
C5	Male	10	5	-1	Negative	34	264	5.8	30	25	3	OFI
C6	Female	6	4	-1	Negative	30	184	3.9	18	30	3.4	OFI
C7	Male	11	6	-1	Negative	35	167	6.7	50	32	3.8	OFI
C8	Female	5	7	-1	Negative	27	106	9.7	90	75	3.2	OFI
C9	Female	5	10	-1	Negative	31	198	4.8	47	29	2.4	OFI
C10	Female	9	5	-1	Negative	37	193	4.1	37	46	3.4	OFI
O1	Female	13	5	-1	Negative	32	165	6.8	58	36	3.3	OFI
O2	Male	8	7	-1	Negative	35	208	2.7	27	33	3.4	OFI
O3	Female	12	5	-1	Negative	35	232	8.2	20	24	4	OFI
O4	Male	6	7	-1	Negative	28	168	9.4	28	30	3.4	OFI
O5	Male	9	7	-1	Negative	34	163	9.1	55	48	3.3	OFI

NA: Not available

Table S2. Demographic and clinical laboratory data of DF patients.

Patient ID	Gender	Age	Day of sample collection		DENV RT-PCR (serotyping)	NS1 levels (ng/ml)	Hematocrit (%)	Platelets (x1,000/ml)	White blood cell count (x1,000 cells/ml)	Aspartate transaminase (AST, U/L)	Alanine transaminase (ALT, U/L)	Albumin (g/dl)	Diagnosis	Classicification
			Based on fever onset	Based on defervescence										
D1	Female	8	4	-1	DENV-1	85	39	155	1.9	135	81	4.1	Secondary DENV infection	DF
D2	Female	8	5	-1	DENV-1	126	34	177	3.4	55	37	3.3	Secondary DENV infection	DF
D3	Male	9	4	-1	DENV-1	513	36	185	1.7	52	36	4	Secondary DENV infection	DF
D4	Female	11	6	-1	DENV-1	1,520	38	75	1.9	42	28	3.9	Secondary DENV infection	DF
D5	Female	11	5	-1	DENV-1	UD	36	96	2.7	147	65	3.9	Secondary DENV infection	DF
F1	Male	13	4	-1	DENV-2	861	43	99	1.5	118	88	3.9	Secondary DENV infection	DF
F2	Male	8	4	-1	DENV-2	932	34	133	3.5	62	37	3.7	Secondary DENV infection	DF
F3	Male	14	5	-1	DENV-2	361	45	77	1.3	84	52	3.5	Secondary DENV infection	DF
F4	Male	13	5	-1	DENV-2	327	35.8	79	2.6	124	37	3.3	Secondary DENV infection	DF
F5	Female	10	5	-1	DENV-2	57	41	175	2.8	37	41	3.7	Secondary DENV infection	DF
E1	Female	11	6	-1	DENV-3	1,247	37.3	51	0.7	229	126	3.1	Secondary DENV infection	DF
E2	Female	9	5	-1	DENV-3	74	37	137	1.8	66	62	3.6	Secondary DENV infection	DF
E3	Female	9	5	-1	DENV-3	364	32	87	2.3	73	37	3.2	Secondary DENV infection	DF
E4	Female	13	5	-1	DENV-3	56	37	82	1.3	33	27	3.4	Secondary DENV infection	DF
E5	Female	10	5	-1	DENV-3	UD	36	106	1.7	54	28	3.5	Secondary DENV infection	DF
B1	Male	9	4	-1	DENV-4	181	37	175	2.6	40	42	3.4	Secondary DENV infection	DF
B2	Female	12	5	-1	DENV-4	510	33	157	2.8	49	36	3.6	Secondary DENV infection	DF
B3	Female	13	6	-1	DENV-4	180	26	134	2.8	49	26	3.3	Secondary DENV infection	DF
B4	Female	10	6	-1	DENV-4	49	34	105	2	104	49	3.5	Secondary DENV infection	DF
B5	Male	8	5	-1	DENV-4	UD	34	405	3.6	62	52	4.7	Secondary DENV infection	DF

UD: Undetectable (below 1 ng/ml)

Table S3. Demographic and clinical laboratory data of DHF patients.

Patient ID	Gender	Age	Day of sample collection		DENV RT-PCR (serotyping)	NS1 levels (ng/ml)	Hematocrit (%)	Platelets (x1,000/ml)	White blood cell count (x1,000 cells/ml)	Aspartate transaminase (AST, U/L)	Alanine transaminase (ALT, U/L)	Albumin (g/dl)	Diagnosis	Classicification
			Based on fever onset	Based on defervescence										
D6	Female	9	4	-1	DENV-1	UD	38	61	1.7	87	35	3.9	Secondary	DHF grade III
D7	Female	11	6	-1	DENV-1	211	39	98	1.5	83	59	3.5	Secondary	DHF grade II
D8	Male	11	6	-1	DENV-1	226	35	103	2.7	119	59	2.8	Secondary	DHF grade II
D9	Female	13	5	-1	DENV-1	595	38	113	1.9	34	33	4.3	Secondary	DHF grade I
D10	Male	11	4	-1	DENV-1	1,516	39	125	3	79	74	3.8	Secondary	DHF grade II
A6	Female	11	6	-1	DENV-2	1,303	37	75	2	100	69	3.7	Secondary	DHF grade I
A7	Male	12	6	-1	DENV-2	681	36	93	2.5	58	42	3.3	Secondary	DHF grade I
A8	Male	12	6	-1	DENV-2	UD	44	20	1.8	152	76	2.9	Secondary	DHF grade III
A9	Male	13	5	-1	DENV-2	240	46	9	4.3	46	43	2.1	Secondary	DHF grade II
A10	Male	10	5	-1	DENV-2	137	40	199	3	91	32	3.8	Secondary	DHF grade II
E6	Male	13	4	-1	DENV-3	1,469	35	49	0.9	58	44	3.6	Secondary	DHF grade I
E7	Male	5	5	-1	DENV-3	144	30.7	121	2.3	67	43	3.3	Secondary	DHF grade I
E8	Male	13	6	-1	DENV-3	608	36	46	5.2	262	60	3.2	Secondary	DHF grade II
E9	Female	12	4	-1	DENV-3	UD	37	57	2.8	452	198	3.7	Secondary	DHF grade I
E10	Female	9	5	-1	DENV-3	319	34	64	3.1	214	81	2.9	Secondary	DHF grade II
B6	Male	11	4	-1	DENV-4	1,524	36	68	1.7	269	110	3.6	Secondary	DHF grade II
B7	Male	12	5	-1	DENV-4	448	35	206	2.3	110	50	3.9	Secondary	DHF grade II
B8	Female	8	5	-1	DENV-4	16	36	32	4.4	125	57	2.9	Secondary	DHF grade III
B9	Female	8	4	-1	DENV-4	35	40	88	2.9	75	71	3.5	Secondary	DHF grade I
B10	Female	11	6	-1	DENV-4	UD	38	32	5.3	111	70	3	Secondary	DHF grade II

UD: Undetectable (below 1 ng/ml)

Table S4. Raman band assignments for SERS spectra of plasma samples.

λ Shift (cm^{-1})	Assignment
644	Uric acid ([1]), Nucleic acid, C-C twisting mode of Tyrosine and phenylalanine ([2])
669	Unknown
702	Tryptophan ([3]), Cholesterol ([2])
724	Adenine ([4])
747	Adenosine diphosphate, hemoglobin ([5])
760	Symmetric breathing of tryptophan ([1])
828	Proline, hydroxyproline, tyrosine, $v_2PO_2^-$ stretch of nucleic acids ([3])
855	Proline, Tyrosine, Tryptophan-IgG ([5])
879	In-plane bending (ring) of deoxyribose (DNA) ([1]), choline, phospholipid ([3])
898	In plane bending of deoxyribose ([4])
904	Glucose (C-O-C skeleton mode) ([6])
938	C–C stretching, protein bands ([6])
980	Unknown
1004	Rocking motions of methyl groups of carotenoids ([6]), symmetric ring breathing mode of phenylalanine ([5])
1032	Phenylalanine-IgG ([5])
1064	Proline (collagen) ([6])
1100	Palmitic acid ([1]), O-P-O backbone stretch of DNA ([3])
1129	C-C skeletal of the acyl backbone in lipids ([6]), Glucose ([1])
1157	C-C (carotenoids) ([5,6])
1177	C-H bending tyrosine ([3])
1208	Amide III-β conformation of protein ([1])
1248	Amide III and CH_2 wagging vibration from glycine backbone and proline side chains ([6])
1284	Amide III ([2])
1320	Guanine ([3])
1417	Urea/triglycerides, CH3 asymmetric deformation ([7])
1449	CH_2-CH_3 deformation (collagen), IgG ([6]), CH_2 bending mode, assigned for fatty acid, lipid and collagen ([5])
1589	C-C stretching of Phenylalanine, Hemoglobin ([5,3])

References

1. Naseer, K.; Amin, A.; Saleem, M.; Qazi, J. Raman Spectroscopy Based Differentiation of Typhoid and Dengue Fever in Infected Human Sera. *Spectrochimica Acta Part A:*

Molecular and Biomolecular Spectroscopy 2019, *206*, 197–201. https://doi.org/10.1016/j.saa.2018.08.008.

2. Saleem, M.; Ali, S.; Bilal, M.; Safdar, K.; Hassan, M. Development of Multivariate Classification Models for the Diagnosis of Dengue Virus Infection. *Photodiagnosis Photodyn Ther* 2022, *40*, 103136. https://doi.org/10.1016/j.pdpdt.2022.103136.

3. Mahmood, T.; Nawaz, H.; Ditta, A.; Majeed, M. I.; Hanif, M. A.; Rashid, N.; Bhatti, H. N.; Nargis, H. F.; Saleem, M.; Bonnier, F.; Byrne, H. J. Raman Spectral Analysis for Rapid Screening of Dengue Infection. *Spectrochim Acta A Mol Biomol Spectrosc* 2018, *200*, 136–142. https://doi.org/10.1016/j.saa.2018.04.018.

4. Gahlaut, S. K.; Savargaonkar, D.; Sharan, C.; Yadav, S.; Mishra, P.; Singh, J. P. SERS Platform for Dengue Diagnosis from Clinical Samples Employing a Hand Held Raman Spectrometer. *Anal. Chem.* 2020, *92* (3), 2527–2534. https://doi.org/10.1021/acs.analchem.9b04129.

5. Khan, S.; Ullah, R.; Saleem, M.; Bilal, M.; Rashid, R.; Khan, I.; Mahmood, A.; Nawaz, M. Raman Spectroscopic Analysis of Dengue Virus Infection in Human Blood Sera. *Optik* 2016, *127* (4), 2086–2088. https://doi.org/10.1016/j.ijleo.2015.11.060.

6. Saleem, M.; Bilal, M.; Anwar, S.; Rehman, A.; Ahmed, M. Optical Diagnosis of Dengue Virus Infection in Human Blood Serum Using Raman Spectroscopy. *Laser Phys. Lett.* 2013, *10* (3), 035602. https://doi.org/10.1088/1612-2011/10/3/035602.

7. Saleem, M.; Ali, S.; Bilal, M.; Safdar, K.; Hassan, M. Development of Multivariate Classification Models for the Diagnosis of Dengue Virus Infection. *Photodiagnosis Photodyn Ther* 2022, *40*, 103136. https://doi.org/10.1016/j.pdpdt.2022.103136.
